# Monocyte‐Derived Macrophages Induce Alveolar Macrophages Death via TNF‐α in Acute Lung Injury

**DOI:** 10.1002/iid3.70081

**Published:** 2024-12-11

**Authors:** Junjie Xiao, Fei Hou, Huan Wang, Ruixuan Wang, Ying Liu, Xiayan Wu, Lixin Xie

**Affiliations:** ^1^ College of Pulmonary & Critical Care Medicine, Chinese PLA General Hospital Beijing China; ^2^ Chinese PLA Medical School Beijing China; ^3^ The 964th Hospital of PLA Joint Logistic Support Force Changchun China; ^4^ State Key Laboratory of Medical Proteomics, Beijing, Proteome Research Center, National Center for Protein Sciences (Beijing), Beijing Institute of Lifeomics Beijing China

**Keywords:** acute lung injury, alveolar macrophages, cell death, monocyte‐derived macrophages, TNF‐α

## Abstract

**Introduction:**

Acute lung injury (ALI) and its subsequent progression to acute respiratory distress syndrome (ARDS) are severe respiratory conditions. They are marked by rapid lung function deterioration and extensive pulmonary inflammation, often resulting in critical patient outcomes. Alveolar macrophages (AMs) and monocyte‐derived macrophages (MDMs) are two distinct subsets of lung macrophages present in the alveoli during ALI. Both are critical mediators of pulmonary inflammation. Our study examined the interplay between AMs and MDMs in the inflammatory environment of ALI/ARDS.

**Methods:**

Mice were treated with lipopolysaccharide (LPS) to establish ALI models. The lung tissues of mice were subjected to hematoxylin‐eosin staining to observe the degree of tissue damage. In vivo, CCR2‐deficient mice or depleting peripheral blood mononuclear cells by clodronate liposomes were used to reduce MDMs recruitment. The bronchoalveolar lavage fluid (BALF) supernatants were used for cytokine and total protein analyses. AMs and MDMs in the BALF were analyzed by flow cytometry. The levels of AMs death were determined through propidium iodide staining and measured by flow cytometry. In vitro, primary AMs were exposed to MDM‐conditioned medium or TNF‐α, and their death levels were assessed under a fluorescence microscope with propidium iodide staining.

**Results:**

AMs significantly decrease in number and undergo extensive cell death during ALI. The reduced MDMs recruitment can increase the number of AMs, reduce AMs death, and alleviate lung injury. In vitro, MDM‐conditioned medium can induce AMs death and TNF‐α is one of the major secretions. It indicates that TNF‐α stimulation in vitro promotes AMs death. In vivo, MDMs are identified as the primary cells secreting TNF‐α. Additionally, the treatment with TNF‐α antagonists can reduce AMs death and the severity of lung injury.

**Conclusion:**

Our study demonstrates that MDMs contribute to AMs death during ALI through TNF‐α. Targeting TNF‐α may offer a therapeutic strategy to mitigate AMs death and lung injury in ALI/ARDS.

## 1 Introduction

Acute lung injury (ALI) is a life‐threatening clinical syndrome characterized by the acute onset of severe hypoxemia and bilateral pulmonary infiltrates without clinical evidence of left atrial hypertension. ALI patients may experience short‐term dyspnea or progress to refractory respiratory failure. In severe cases, ALI can lead to acute respiratory distress syndrome (ARDS), which is a major cause of morbidity and mortality [1, 2]. Current treatments of ALI/ARDS are primarily supportive and focus on the underlying condition and bedside care, including mechanical ventilation and corticosteroid administration. These treatments have improved outcomes, yet mortality rates can exceed 40% [3]. The Corona Virus Disease 2019 (COVID‐19) pandemic has significantly impacted lives, with ARDS as a primary mortality cause. Approximately one‐third of hospitalized COVID‐19 patients developed ARDS, with a mortality rate of about 70% [4].

ALI/ARDS can stem from direct lung injury (like pneumonia or gastric aspiration) or indirect systemic inflammatory responses (such as sepsis, trauma, or major surgery). Recently, the prevalence of traumatic injury‐related ARDS has decreased due to changes in mechanical ventilation, crystalloid resuscitation, and transfusion strategies. Meanwhile, vaping‐associated lung injury, drug‐induced ARDS, and viral pneumonia have emerged as increasing ARDS causes [5]. Diffuse alveolar injury, characterized by hyaline membranes forming along the denuded alveolar basement membrane in substantial epithelial lung injury areas, is the dominant pathological ALI feature [6]. The pathophysiology of ALI is complex, involving factors like the inflammatory response, oxidative stress, increased vascular permeability, coagulation dysfunction, and an abnormal immune response [7]. These activities aiming to combat infection or injury often lead to uncontrolled inflammation, both pulmonary and systemic. A leading theory for ALI development is cell death, which contributes a lot to the uncontrolled production and secretion of pro‐inflammatory cytokines [8]. A range of damage‐associated molecular patterns (DAMPs), such as IL‐1a, IL‐1b, IL‐18, IL‐33, HMGB1, galectin‐1, or DNA, are released following cell death, driving tissue inflammation and cell death pathways [9]. These DAMPs can drive both tissue inflammation and the activation of further pathways of regulated cell death. Following an initial event of regulated necrosis, cell death and inflammation can induce each other and drive a local auto‐amplification loop that leads to exaggerated cell death and inflammation [10]. Cell death has been observed in the lungs and other organs during ALI/ARDS pathogenesis [11, 12]. ARDS and systemic inflammatory response syndrome (SIRS) are considered as serious consequences of overwhelming inflammation related to cell death [13].

Alveolar macrophages (AMs), comprising about 95% of airspace leukocytes, are specialized macrophages in lung alveoli and the first line of immune defense in the respiratory tract [14]. AMs initiate lung inflammation as innate immune cells. Their death can exacerbate lung injury, which is an important component of the cell death and inflammatory response amplification cycle. Recent evidence suggests that AMs cell death significantly influences the progression of lung inflammation [15, 16, 17]. Understanding how AMs death affects lung inflammation is crucial for comprehending ALI/ARDS mechanisms.

After pathogens invade the lungs, AMs activate rapidly, and neutrophils are recruited in large numbers. Monocytes from the bone marrow migrate to the lung tissue in a CCL2/CCR2 axis‐dependent manner, differentiating into macrophages [18]. These recruited macrophages, known as monocyte‐derived macrophages (MDM), increase significantly in number during acute lung injury [19]. MDMs can produce various pro‐inflammatory and anti‐inflammatory mediators to regulate local inflammatory responses in the lungs. However, these recruited macrophages differ from AMs because they secrete more inflammatory factors and have a more pro‐inflammatory profile [20].

Tumor necrosis factor‐alpha (TNF‐α) plays a pivotal role in immune regulation and inflammation. As part of the TNF superfamily, it is primarily produced by activated macrophages, T cells, and NK cells [21]. TNF‐α effects are mediated through binding to two receptors, TNFR1 (p55) and TNFR2 (p75), which are expressed on various cells. Its binding to TNFR1 primarily activates pro‐inflammatory pathways, leading to inflammation, cell proliferation, and survival gene transcription. This interaction can also induce apoptosis via a death‐inducing signaling complex. Balancing these pathways is essential for immune response regulation and cell fate [22, 23, 24].

Here, we report that monocyte‐derived macrophages contribute significantly to AMs death via TNF‐α during ALI phases. CCR2^−/−^ mice showed protection from AMs loss and reduced lung injury. Blocking TNF‐α in vivo reduced dead AMs in the lungs and lung tissue damage, highlighting the role of MDM‐secreted TNF‐α in AMs death.

## 2 Materials and Methods

### 2.1 Mice

C57BL/6 wild‐type (WT) mice were purchased from Charles River in Beijing (Vital River). CCR2^+/−^ and CCR2^−/−^ (B6.129S4‐Ccr2tm1Ifc/J) mice were purchased from Jackson Laboratory. All mice (*n* = 40, 8 weeks old, 20–25 g) were kept in groups of five in a standard laboratory at the Beijing Institute of Lifeomics. The room had a temperature of 23 ± 1°C, humidity at 55% ± 5%, and a 12‐h light‐dark cycle, with free access to food and water. Experimental protocols were approved by the Animal Care and Use Committee of the university (IACUC‐20200221‐02MO). All possible efforts were made to improve animal welfare and minimize the use of animals in the study.

### 2.2 Groups and LPS‐Induced ALI Model

The study's primary supervisor, responsible for assigning and conducting the experiment, knew about the various experimental groups. As a result, none of the individuals involved in data collection and analysis were informed about group allocation. We opted for a limited sample size based on previous research findings, which would be sufficient to detect significant differences between groups in the primary outcome. The number of mice used in each group is detailed in the figure legend.

Mice were anesthetized before LPS instillation with 2.5% avertin (20 mL/kg, intraperitoneal (i.p.); MilliporeSigma, USA). Using a 24‐gauge catheter, *Escherichia coli* LPS (O111:B4 Sigma‐Aldrich L2630) at 2 μg/g mouse, or PBS as a control, was intratracheally instilled in mice. Cages were assigned a numerical designation based on their position on the rack. On Ddays 1, 3, and 5 postinstillations, a cage was randomly selected from each group, mice were anesthetized and then euthanized by exsanguination via the inferior vena cava following humane endpoints. The testing order of each animal within each group was randomized. The modeling and measurement methods employed in this study are based on the established approaches published in earlier research. The data was included in the study if the mice underwent successful measurement of ALI. The data was excluded if the mice died during the measurement.

### 2.3 Bronchoalveolar Lavage Fluid (BALF) Collection

After anesthesia, the tracheas were cannulated with a 20‐gauge catheter. The mice lungs were washed three times with 0.8 M chilled PBS containing 5 mM EDTA (PBE). In some cases, the left lung was ligated to determine the dry‐to‐wet ratio or hematoxylin‐eosin (H&E) staining, while the right lung was lavaged three times with 600 μL of PBE. The collected BALF was centrifuged at 500 g for 5 min at 4°C. The obtained pellets were resuspended in PBE and subjected to flow cytometry analysis. Meanwhile, supernatants were collected for cytokine and total protein analysis.

### 2.4 Flow Cytometry and Cell Sorting

Before staining with specific antibodies, cells were pretreated with Fc Block‐2.4G2 (70‐0161) to inhibit Fcγ III/II receptors. For cell surface marker analysis, cell pellets were stained with appropriate antibodies at 4°C for 20–30 min. For intracellular cytokine analysis, we used the Cytofix/Cytoperm kit (eBioscience, USA) following the kit's instructions. The following antibodies were used for staining: CD45‐AF700 (30‐F11), Ly6G‐PEcy7 (1A8), CD64‐AF646 (X54‐5/7.1), MerTK‐BV711 (DS5MMER), Siglec‐F‐BV421 (E50‐2440), CD11b‐BUV395 (M1/70), CD3‐FITC (145‐2C11), CD11c‐PEcy(M418), NK1.1‐BV510(PK136), EpCAM‐APC (G8.8), CD31‐FITC (390), TNF‐a‐PE(MP6‐XT22), CD115‐BV605(AFS98). To estimate the percentage of cell death, cells were subjected to propidium iodide staining (Invitrogen, 2481410) in a cytometry buffer for 15 min on ice in the dark. Flow cytometry was performed using an LSR II Fortessa system (BD Biosciences, USA). The acquired data were analyzed with the FlowJo software (Tree Star). For cell sorting, we used FACS Aria III (BD Biosciences, USA).

### 2.5 BALF Analysis

The BALF supernatants were used for cytokine and total protein analysis. Total protein was measured by the bicinchoninic acid method. Cytokines were measured using the magEasyQPlex mouse 10‐plex Flow Assay Kit (PLEM100, Laizee Biotech, Shanghai) following the manufacturer's instructions.

### 2.6 Wet‐To‐Dry Ratio of Lung

After inducing lung injury with LPS, the left lung lobe was removed, weighed, and then dried for 24 h at 55°C to calculate the wet‐to‐dry ratio of the lung.

### 2.7 Histopathological Analysis

The lungs were inflated and preserved in 10% formalin for 48 h and then embedded in paraffin. Thin tissue sections of 5 μm thickness were cut, stained with hematoxylin and eosin (H&E), and then imaged under a Leica Aperio slide scanner (Leica, Wetzlar, Germany).

### 2.8 AMS Isolation

AMs were collected from BAL by flushing the lungs three times with 0.8 mL of chilled PBS containing 5 mM EDTA. The lavage was repeated three times, and AMs were pooled from several mice. Cells were seeded at 3 × 105/well in RPMI‐1640 medium (EallBio, Germany) for 2 h. Nonadherent cells were removed by washing, and the adherent cells were AMs. The purity of isolated AMs was above 90%.

### 2.9 AMS Stimulation and Cell Death Analysis

AMs (3 × 105 cells/well) were seeded in 48‐well tissue culture plates. Cells were treated with the MDM‐conditioned medium, 200 ng/mL of TNF‐α (Peprotech, 061454‐1, USA) or 200 ng/mL of IL‐6 (Peprotech, 216‐16, USA) and stained with 2.5 µg/mL of propidium iodide (Invitrogen, 2549278, USA). The plate was scanned for fluorescent and phase‐contrast images (four image fields/well) after 24, 48, and 72 h under an inverted fluorescence microscope (Nikon Tie, Japan). The image analysis was done using the software package supplied with the microscope. and quantification of dead cells was done by Image J software.

### 2.10 Monocyte Depletion

Monocytes were depleted by injecting 200 µL clodronate liposomes (LIPOSOMA, Netherlands) or PBS liposomes as a control via the tail vein. The mice were numbered before administration and then randomly divided into clodronate liposomes group and control group using a random number table.

### 2.11 Peripheral Blood Mononuclear Cell Analysis

Peripheral blood was collected from the mouse orbital cavity using a collection tube containing the anticoagulant EDTA. After lysing with 1 mL of red blood cell lysis buffer on ice for 3 min, the cells were washed with 1 mL of PBE solution. Following centrifugation at 500 G for 5 min at 4°C, the cell pellet was collected for subsequent antibody labeling.

### 2.12 Neutralizing Antibody Treatment

Age‐ and gender‐matched 6‐ to 8‐week‐old WT mice were administered 200 μg of Infliximab (i.p., TargetMoI, T992, USA) in PBS or PBS alone as a control at 24, 48, and 72 h after intratracheal injection of LPS. Mice were monitored for survival and weight change.

### 2.13 Statistical Analysis

Statistical analysis was performed with GraphPad Prism (v8) software. Data are presented as mean ± SEM. Sample data with a normal distribution were analyzed using the Student's *t*‐test as appropriate.

## 3 Results

### 3.1 AMS Undergo Cell Death and Diminish in Number During ALI

**Figure 1:**
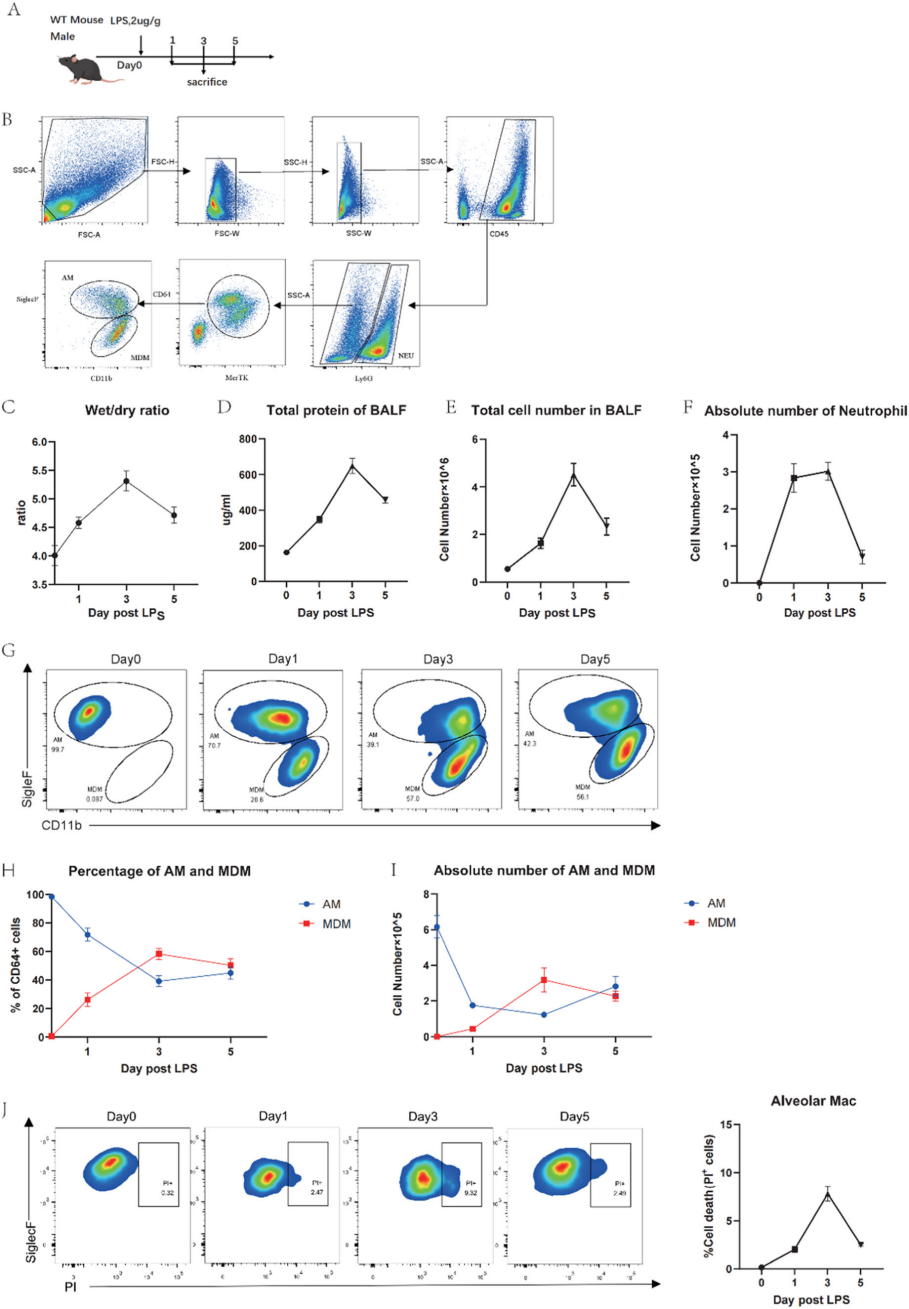
The dynamic changes of AMs and MDMs in the BALF of ALI mice. (A) mice were sacrificed on days 1, 3, and 5 after intratracheal instillation of LPS (2 μg/g mouse), and materials were also obtained from untreated healthy mice (n = 4 per time point). (B) Gating strategy of AMs and MDMs in BALF cells. AMs were identified as CD45^+^Ly6G^−^CD64^+^MerTK^+^SiglecF^+^CD11b^−^ cells; MDMs were identified as CD45^+^Ly6G^−^CD64^+^MerTK^+^SiglecF^−^CD11b^+^ cells. (C) Wet/dry weight ratio changes of lung tissue. (D) Total protein level and (E) total cell number in ALI mice BALF. (F) Absolute number of neutrophils in BALF at each time point. (G) Flow cytometry of AMs and MDMs in BALF and (H) their proportion and (I) quantity at each time point. (J) The death proportion of AMs at each time point. Data are presented as mean ± SEM.

To explore the dynamics of AMs and MDMs in ALI, mice were injected with LPS intratracheally (Figure 1A). AMs and MDMs were distinguished by SiglecF and CD11b expression levels using the gating strategy shown in Figure 1B. After LPS administration, the wet‐to‐dry ratio of the mouse lungs, as well as the total protein concentration, total cell number and absolute number of neutrophils in BALF all peaked on the third day and declined by the fifth day (Figure 1C–F). These data suggest that the inflammatory response in the lungs of mice reached its peak around the third day and began to subside by the fifth day in this model. During this period, MDMs were extensively recruited, increasing both in proportion and absolute quantity among CD64^+^ pulmonary macrophages on Day 3 (Figure 1G–I). Meanwhile, AMs not only decreased in proportion but also in number, reaching a low on Day 3 (Figure 1I). The AMs death rate corresponded to these changes, peaking on the third day (Figure 1J).

### 3.2 MDMS Play a Significant Role in the Reduction and Death of AMS

**Figure 2:**
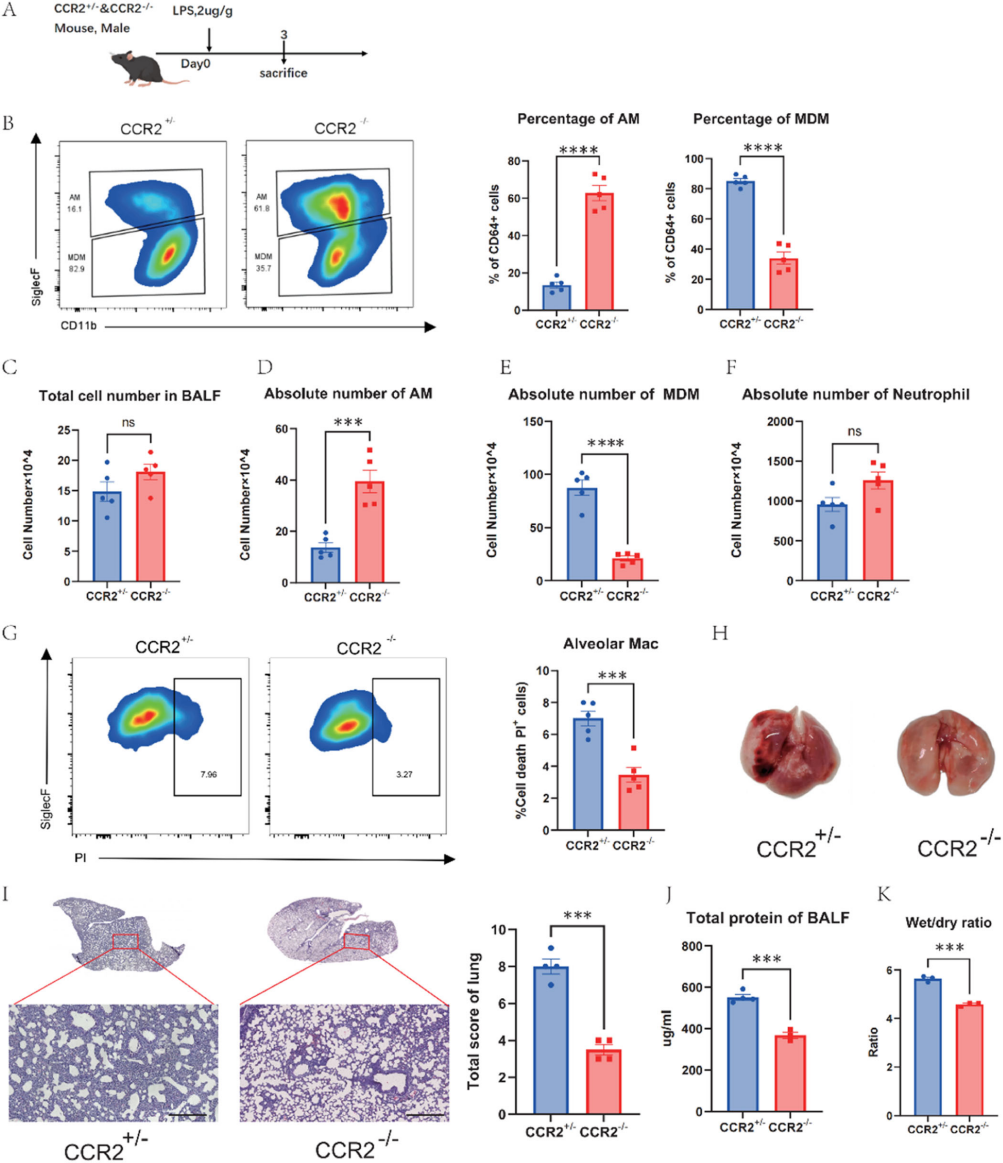
Reduced AMs death and alleviation of lung injury of CCR2^−/−^ mice correlated to decreased recruitment of MDMs. (A) CCR2^−/−^ and CCR2^+/−^ mice were sacrificed after intratracheal LPS instillation (2 μg/g mouse) for 3 days (n = 5). (B) Flow cytometry of AMs and MDMs in BALF and their proportion. (C) Total cell number in BALF and the absolute number of (D) AMs, (E)MDMs and (F) neutrophils. (G) The death proportion of AMs. (H) The outlook of lung tissue at day3 post LPS. (I) H&E staining and pathological scores of the left lobe of CCR2^−/−^ and CCR2^+/−^ mice. Scale bars, 100 µm (J) Total protein level in mice BALF. (K) Wet/dry weight ratio of lung tissue. Data are presented as mean ± SEM. ns, not significant, ***P < 0.001 and ****P < 0.0001. Analysis was performed using Student's t‐test.

The shifts in AMs and MDMs populations suggest a link between AMs death in ALI and MDMs migration. To test this, CCR2‐deficient mice were used (Figure 2A). The absence of CCR2 resulted in a reduction in the recruitment of MDMs but does not significantly affect the recruitment of neutrophils (Figure 2B,E,F). In these mice, MDMs recruitment was significantly reduced, as shown by both proportion and absolute numbers (Figure 2B,E). Consequently, AMs rebounded in both proportion and quantity compared to control mice (Figure 2D). Analysis showed a significant reduction in AMs cell death in the CCR2 knockout group (Figure 2G). Additionally, lung injury extent was notably mitigated in these mice. The hemorrhagic spots in the lung tissue were significantly reduced (Figure 2H), and the wet‐to‐dry weight ratio decreased (Figure 2K). In the knockout group, histopathological sections of mouse lung tissue stained with H&E showed reduced infiltration of inflammatory cells and a decrease in the thickness of the alveolar walls (Figure 2I). A notable reduction in the total protein concentration in the lavage fluid was also detected (Figure 2J). These results suggest that blocking MDMs recruitment reduces AMs death and lung injury, implicating MDMs play a significant role in early AMs death during ALI.

**Figure 3:**
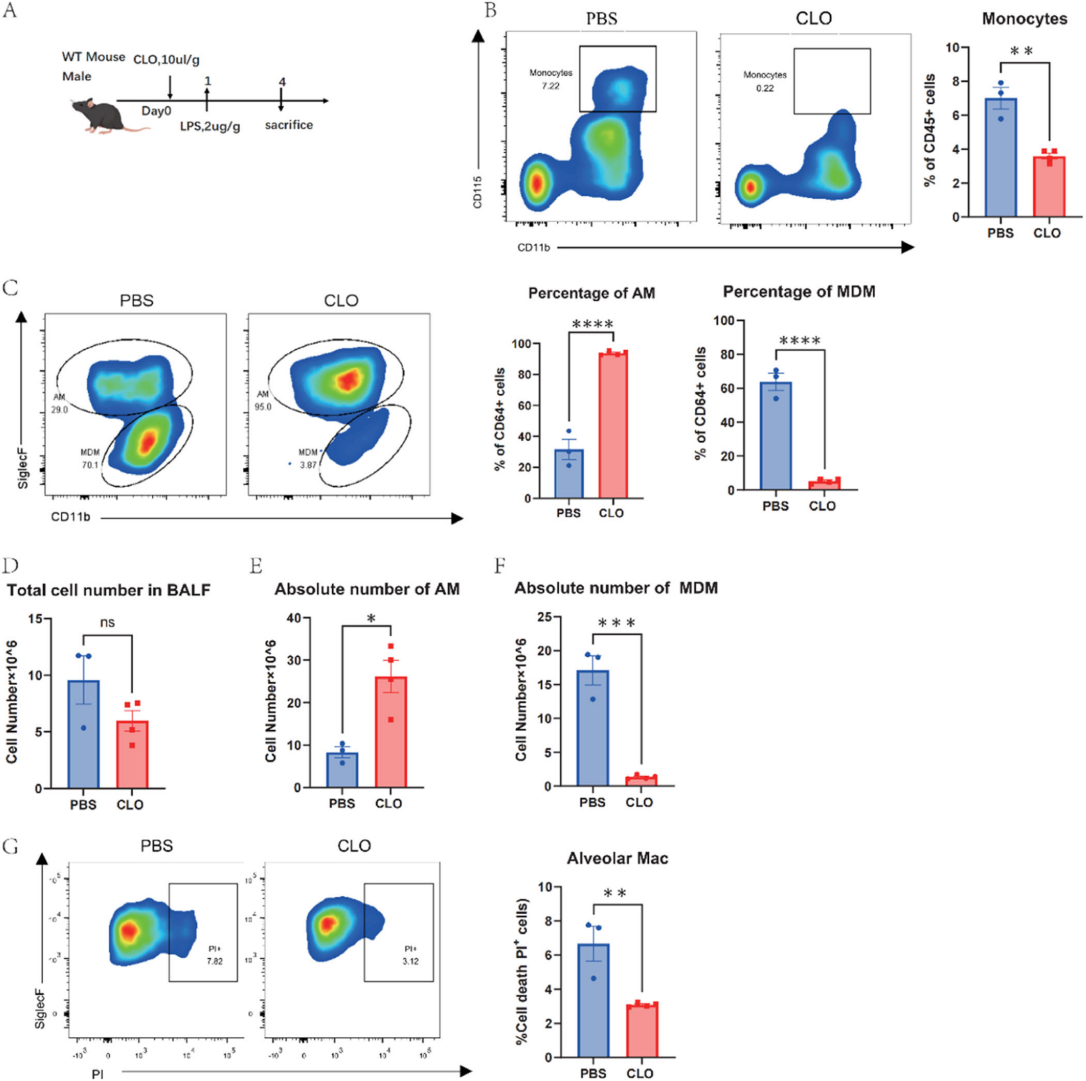
Reduced AMs death after peripheral monocyte clearance with chlorophospholipid liposomes. (A) Wild‐ type mice were injected 200ul clodronate liposomes or PBS liposomes as a control via the tail vein one day before the intratracheal LPS instillation (2 μg/g mouse), mice were sacrificed on days 3 post‐ LPS (n = 3). (B) The clearance efficiency of peripheral blood monocytes on day 4. (C) Flow cytometry of AMs and MDMs in BALF and their proportion. (D) Total cell number in BALF and absolute number of (E) AMs, (F) MDMs. (G) The death proportion of AMs. Data are presented as mean ± SEM. ns, not significant, *P < 0.05, **P < 0.01, ***P < 0.001 and ****P < 0.0001. Analysis was performed using Student's t‐test.

To further support our findings, a mouse model with depleted peripheral blood mononuclear cells was created using clodronate liposomes (CLO) administered via tail vein injection. ALI was then induced with LPS (Figure 3A). We observed effective monocyte depletion (Figure 3B), inhibiting MDMs recruitment to lung injury sites (Figure 3C,F). Also, a significant decrease in AMs death and a restoration of AMs quantities were noted (Figure 3C,E).

### 3.3 Secretions from MDMS Induce the Death of AMS

**Figure 4:**
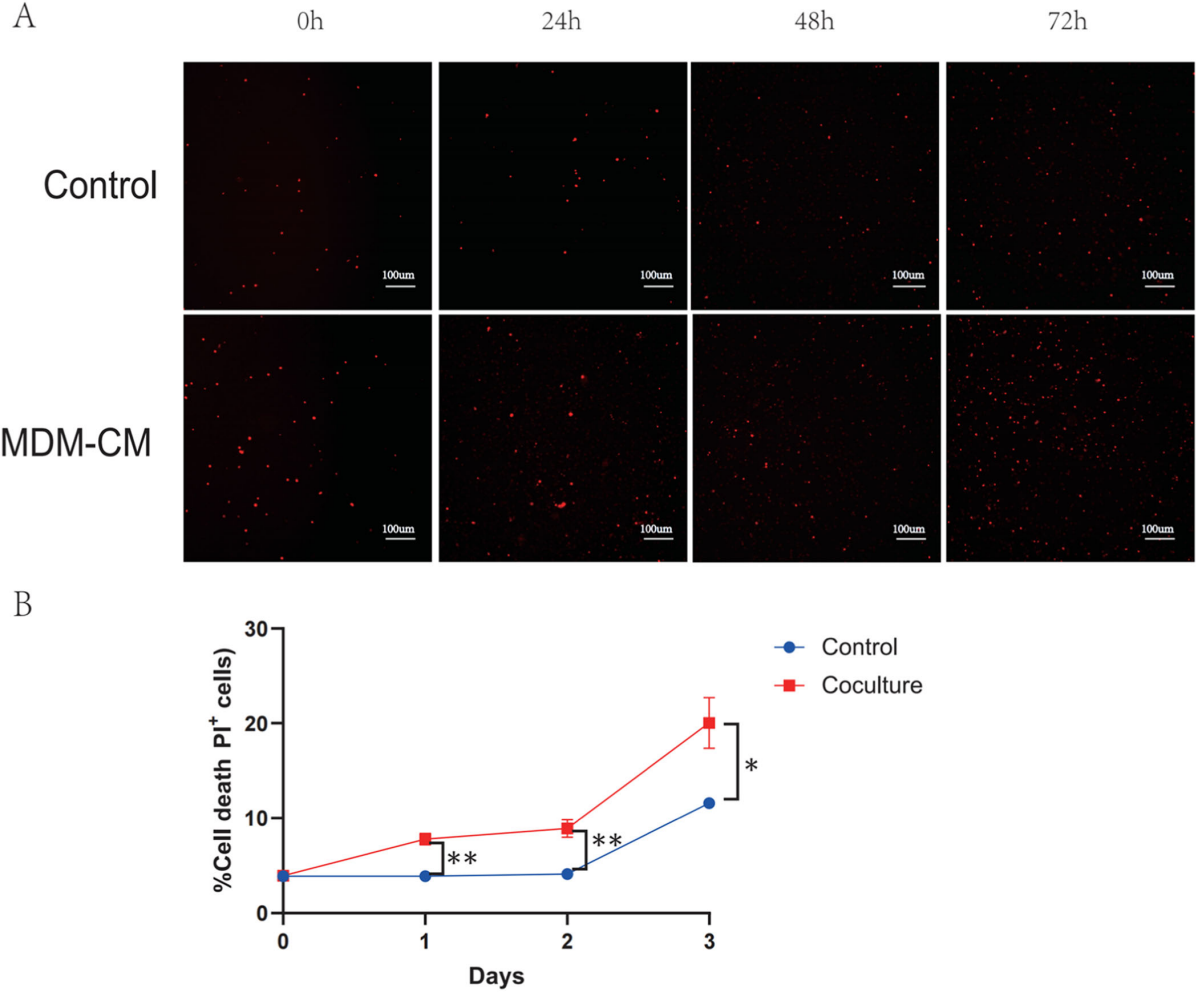
Indirect coculture of AMs and MDMs. (A) Representative images of cell death in AMs treated with MDM‐CM after 0h, 24h, 48h and 72h. AMs (3 × 105 cells/well) were seeded in 48‐well tissue culture plates. Cells were treated with MDM‐conditioned medium (MDM‐CM) and stained with 2.5 µg/ml of propidium iodide. The plate was scanned for fluorescent and phase‐contrast images (4 image fields/well) at each time point. Scale bar, 100 µm. (B) Analysis of cell death in AMs with the indicated treatments (n = 4 per time point). Data are presented as mean ± SEM. ns, not significant, *P < 0.05, **P < 0.01. Analysis was performed using Student's t‐test.

Upon confirming the crucial role of MDMs in AMs cell death during the early‐stage of ALI, we focused on the underlying mechanism. We hypothesized that MDM secretory products might be involved. To investigate this, MDMs mobilized in the acute phase of ALI in WT mice were isolated and cultured ex vivo for 24 h to obtain a supernatant enriched with MDM‐derived secretions. This MDM‐conditioned medium (MDM‐CM) was used to treat normal murine AMs. Time‐lapse fluorescence imaging for AMs viability assessment showed a marked increase in cell death among AMs exposed to MDM‐derived secretions compared to the control group. And the disparity grew over time (Figure 4A,B). These findings suggest that MDMs predominantly precipitate AMs death in ALI through their secretory products.

### 3.4 TNF‐α is Crucial in MDM‐Induced AMS Death

**Figure 5:**
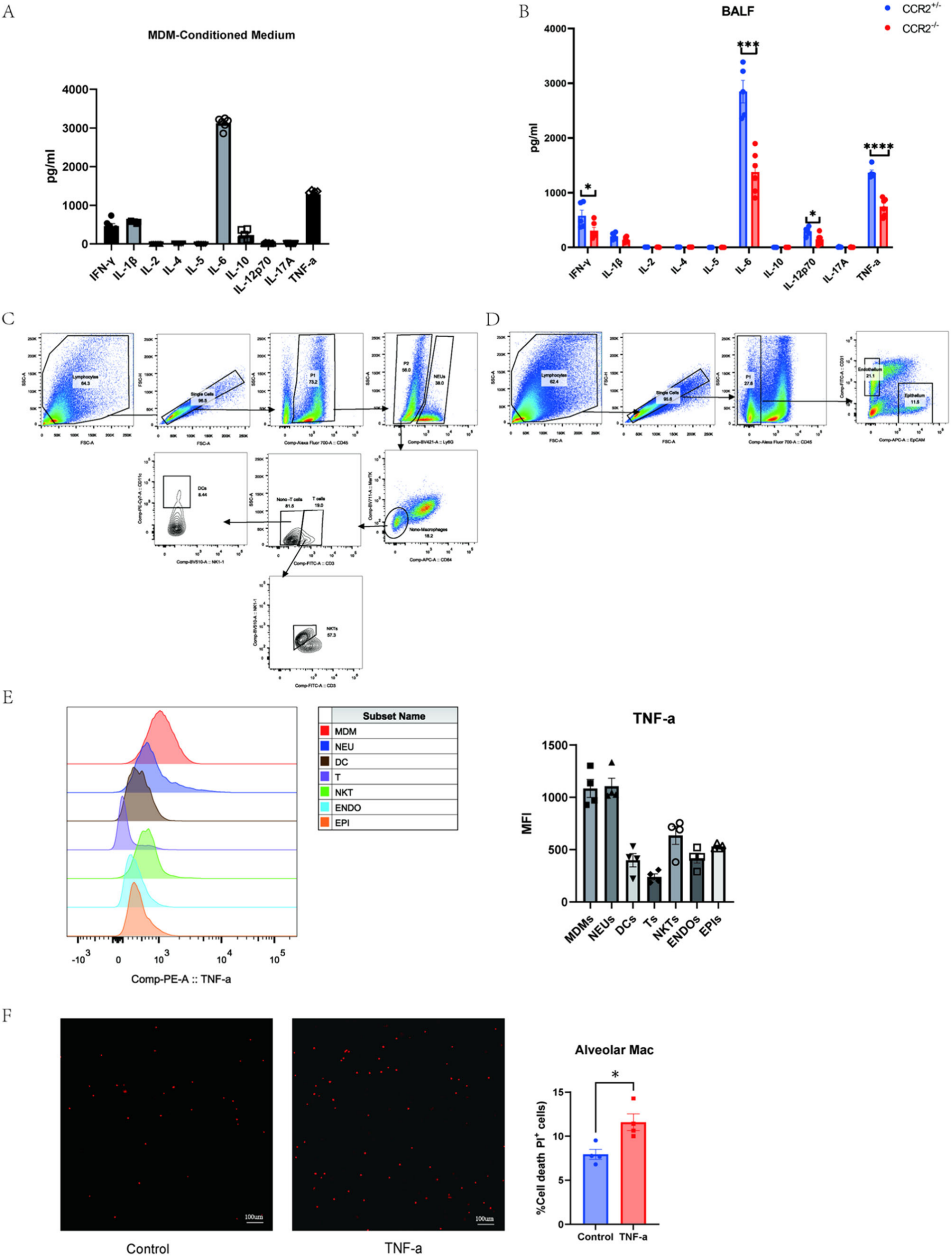
TNF‐α is crucial in MDM‐induced AMs death. (A) Multicellular cytokines levels in MDM‐conditioned medium (n = 6). (B) Multicellular cytokines levels in the BALF of CCR2^−/−^ and CCR2^+/−^ mice on day 3 of ALI (n = 5). (C‐D) Gating strategy of neutrophils (NEUs), dendritic cells (DCs), T cells (Ts), natural killer T cells (NKTs), epithelial cells (EPIs) and endothelial cells (ENDOs). (E) Mean Fluorescence Intensity of TNF‐α expression levels (n = 3). (F) Representative images of cell death in AMs treated with TNF‐α (200 ng /mL) after 48 h. Scale bar,100 µm. Data are presented as mean ± SEM. ns, not significant, *P < 0.05, **P < 0.01, ***P < 0.001 and ****P < 0.0001. Analysis was performed using Student's t‐test.

MDMs are inflammatory cells recruited and differentiated from peripheral monocytes. During acute inflammation, MDMs primarily polarize towards an M1 phenotype. M1‐type MDMs secrete cytokines like TNF‐α, IFN‐γ, IL‐1, and IL‐6, which are key in mediating inflammation. In this study, we conducted multiple cytokine concentration assays on MDM‐conditioned medium. The results showed that among the 10 cytokines tested, IL‐6 and TNF‐α were the primary secretory factors of MDMs (Figure 5A). Additionally, BALF cytokine levels in CCR2^+/^^−^ and CCR2^−/−^ mice showed a significant decrease in IL‐6 and TNF‐α levels in CCR2^−/−^ mice, correlating with reduced MDMs recruitment in ALI (Figure 5B). Compared to IL‐6, TNF‐α is known to induce cell death, driving inflammatory responses [25]. Thus, we consider TNF‐α a potential key factor in MDM‐induced AMs cell death.

In fact, many cell types secrete TNF‐α as a central mediator in ALI. We used an intracellular antibody against TNF‐α and flow cytometry to assess TNF‐α levels in different cell types (Figure 5C,D). Our results indicated that MDMs and neutrophils are primary TNF‐α producers (Figure 5E). Stimulating primary murine AMs in vitro with recombinant mouse TNF‐α confirmed that TNF‐α can induce AMs cell death (Figure 5F), supporting our hypothesis. Moreover, IL IL‐6, unlike TNF‐α, does not induce AMs death, and the costimulation of TNF‐α and IL‐6 does not increase the level of cell death compared to TNF‐α alone (Supporting Information S1: Figure S1). These findings suggest that while IL‐6 is a significant inflammatory molecule, its contribution to AMs death can be excluded in the context of our experiments. In summary, TNF‐α is a key factor in MDM‐promoted AMs cell death.

### 3.5 Blocking TNF‐α Reduces the Death of AMS and Alleviates Lung Injury

**Figure 6:**
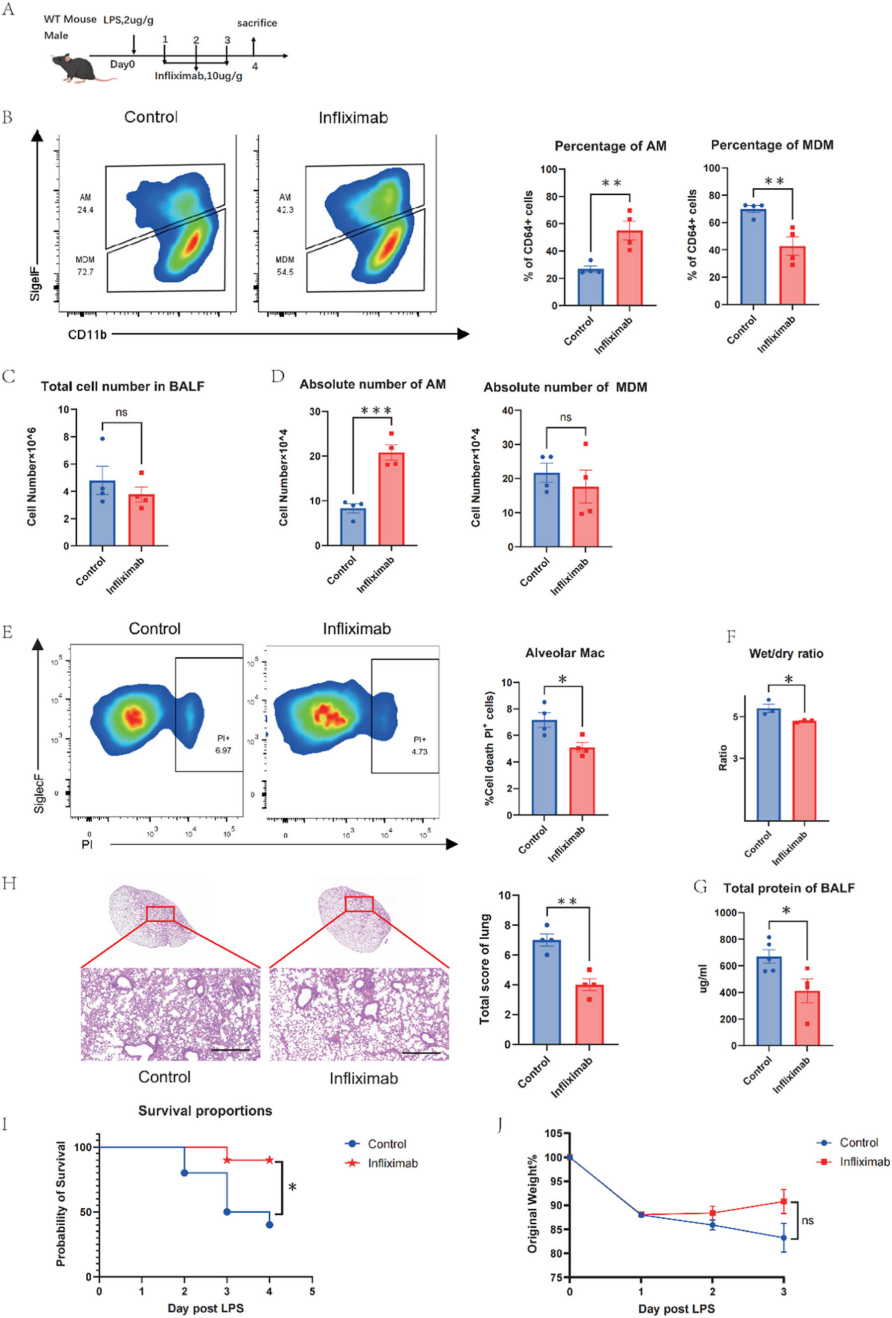
Blocking TNF‐α reduces the death of AMs and alleviates lung injury. (A) Mice were administered infliximab (10 μg/g mouse) intraperitoneally at 24,48,72 h after intratracheal injection of LPS (2 μg/g mouse), mice were sacrificed on days 4 (n = 4). (B) Flow cytometry of AMs and MDMs in BALF and their proportion. (C) Total cell number and (D) absolute number of AMs and MDMs in the BALF. (E) The death proportion of AMs. (F) Wet/dry weight ratio of lung tissue. (G) Total protein level in mice BALF. (H) H&E staining and pathological scores of the left lobe of mice. Scale bars, 100 µm. (I) survival and (J) the change of weight were monitored. Data are presented as mean ± SEM. ns, not significant, *P < 0.05, **P < 0.01, ***P < 0.001. Analysis was performed using Student's t‐test.

Based on our findings that MDM‐derived TNF‐α drives AMs inflammatory cell death, we hypothesized that inhibiting TNF‐α would reduce AMs death in vivo, protecting against LPS‐induced lung injury. Mice were treated with infliximab, a chimeric (mouse/human) antibody cross‐reacting with murine TNF, after intratracheal LPS injection (Figure 6A). Infliximab‐treated mice showed lower AMs cell death rates, with recovery in their population and absolute numbers (Figure 6B,D,E). The treatment group showed a significant reduction in lung injury severity, as evidenced by a decrease in the lung wet‐to‐dry weight ratio (Figure 6F), reduced total protein content in the BALF (Figure 6G), and a decrease in inflammatory cell infiltration in the lung tissue (Figure 6H). Moreover, these mice displayed significantly lower mortality rates compared to the control group (Figure 5I). Although no statistical difference was observed, there was a slight recovery in the body weight of mice in the treatment group (Figure 5J). In conclusion, these findings of the study demonstrate that TNF‐α inhibition with infliximab can reduce the death of AMs and alleviate LPS‐induced lung injury in mice.

## 4 Discussion

During ALI, like neutrophils, MDMs are rapidly recruited to the lungs in large numbers in the early phase of inflammation. While the mechanisms by which neutrophils' overactivation promotes the development of ALI have been relatively clear, the role of MDMs in ALI has also increasingly gained research attention in recent years. Previous studies have suggested that these peripherally recruited MDMs, unlike tissue‐resident AMs, exhibit higher pro‐inflammatory cytokine levels during the inflammation peak [26]. Liao Ming and his colleagues conducted a single‐cell RNA sequencing analysis of BALF collected from severe or mild COVID‐19 patients. They revealed that monocytes accounted for 80% of the total BALF cells in severe COVID cases, compared to approximately 60% and 40% in mild disease patients and healthy controls, respectively. Further characterization of the MDMs composition showed a depletion of tissue‐resident alveolar macrophages and an enrichment of inflammatory MDMs in patients with severe disease [27]. Consistently, our research model demonstrated a similar pattern, with a notable early recruitment of MDMs and a correspondingly significant reduction in AMs. Moreover, our experiments utilizing CCR2 knockout mice provided compelling insights. We discovered that genetic ablation of CCR2 effectively curtailed excessive MDM recruitment to the lungs. This intervention mitigated extensive AMs cell death and notably ameliorated overall lung injury. These observations underscore the critical and non‐negligible role of MDMs in the pathophysiology of ALI, offering new perspectives on the cellular interactions driving this complex condition.

During IAV infection, impairment of the AMs response leads to both uncontrolled IAV replication and pathogenesis. Depleting AMs or disrupting the maturation of AMs is associated with pathological alterations and lethality in IAV‐infected mice [28]. Research has indicated that AMs can be directly stimulated by pathogens or critical signal molecules to activate their death pathways. Additionally, research focusing on how interactions between other immune cells and AMs lead to their death has emerged as a hot topic. Peiró et al. conducted research suggesting that the interaction between AMs and neutrophils, mediated by the antibacterial peptide LL‐37 secreted by neutrophils, can lead to the activation of pyroptotic pathways in AMs [29]. Jiao Y et al. discovered that neutrophil‐secreted exosomes transfer miR‐30d‐5p to macrophages, which activates the NF‐κB signaling pathway and triggers pyroptosis in AMs by activating NLRP3 [30]. Lee and Pan showed that HMGB1 in NETs activates RAGE, which in turn activates caspase‐1, promoting pyroptosis in AMs and exacerbating lung injury [31]. Moreover, Li Na and her colleagues found that NKT cells are the major source of pro‐inflammatory LIGHT in the pathogenesis of influenza pneumonia. They serve as the proapoptotic signal for LTbR+ AMs [32]. Hence, the death of AMs cannot be fully explained by direct pathogen stimulation alone, as other immune cells also play complex roles. Our study elucidates that MDMs significantly contribute to the inflammatory death of AMs in ALI and that clearing or reducing the recruitment of MDMs can effectively decrease AMs death. Among various inflammatory substances secreted by MDMs, we identified TNF‐α as the core molecule promoting AMs death. Notably, MDMs and neutrophils are the primary contributors to TNF‐a. Although other types of cells can also secrete some TNF‐α, their contribution is far less significant.

TNF‐α is a prominent member of the TNF family. This family of cytokines, characterized by similar structural domains, plays a key role in regulating cellular processes such as survival, proliferation, and death. TNF‐α is known to activate the expression of inflammatory genes through the NF‐κB and MAPK pathways and can also induce various forms of cell death, including apoptosis, necroptosis, and pyroptosis. The biological effects of TNF‐α are mediated through its interaction with two primary receptors, TNFR1 and TNFR2. Activation of TNFR1 is closely associated with the induction of cell death, whereas TNFR2, lacking a death domain, is involved in promoting cell survival and anti‐inflammatory responses. The death domain in TNFR1 interacts with the TNF receptor‐associated death domain protein, leading to the formation of a primary signaling complex (Complex I). This complex activates the MAPK and NF‐κB pathways, which in turn induce the expression of pro‐inflammatory genes. However, cell death in response to TNF‐α is not an automatic outcome. Instead, it is tightly regulated by several cell death checkpoints that prevent excessive cytotoxicity. The three primary cell death checkpoints in the TNFR1 pathway are the IKK, NF‐κB, and caspase‐8 checkpoint. The cell death response triggered by TNFR1 proceeds only when one of these checkpoints is inactivated. Inactivation of the IKK checkpoint results in the activation of RIPK1 within Complex I and the subsequent assembly of a secondary cytosolic complex (Complex IIb). Depending on the cellular environment, it can lead to either RIPK1 kinase activity‐dependent apoptosis by the activation of caspase 3/7 or pyroptosis following the cleavage of GSDMD. When the NF‐κB checkpoint is disrupted, it can lead to RIPK1 kinase activity‐independent apoptosis through the activation of Complex IIa. Furthermore, the inactivation of the caspase‐8 checkpoint leads to RIPK1 kinase activity‐dependent apoptosis. In conditions where caspase‐8 is inhibited, RIPK1 interacts with RIPK3 to form a necrosome (Complex IIc), which activates MLKL and leads exclusively to necroptosis [25, 33].

Cell death, occurring in various forms, is recognized as a host defense mechanism, aiming to eradicate pathogens by depriving them of their replication environment and signaling the immune system to initiate a response. However, cell death induced by TNF is not always advantageous for the host. In some contexts, excessive activation of TNF‐mediated cell death pathways can exacerbate, rather than mitigate, the pathogenicity and lethality of microorganisms. This phenomenon is particularly evident in infections caused by severe acute respiratory syndrome coronavirus 2, Mycobacterium tuberculosis, and Bacillus anthracis, as reported in several studies [13, 34, 35]. TNF‐α is involved in various physiological processes and pathological conditions, including infectious and inflammatory diseases, autoimmune diseases, and cancers. Due to its central role in inflammation and cell death, it has been a target for therapeutic interventions in diseases like rheumatoid arthritis and inflammatory bowel disease, where TNF inhibitors are used to reduce inflammation and tissue damage.

The clinical success of anti‐TNF biologics in treating inflammatory diseases is due to their effectiveness in blocking the interaction of TNF with its receptors, TNFR1 and TNFR2. Traditionally, this blockade is thought to alleviate inflammation by preventing the activation of the mitogen‐activated protein kinase (MAPK) pathway and the nuclear factor kappa‐light‐chain‐enhancer of activated B cells (NF‐κB) pathway. These pathways would otherwise lead to the upregulation of pro‐inflammatory genes, a key process in the pathophysiology of inflammation [25]. Our study elucidates the critical role of TNF‐α in MDM‐mediated promotion of AMs death and attempts to use TNF‐α antibodies as a therapeutic intervention. These antibodies aim to mitigate the extensive death of AMs, thereby alleviating or treating ALI. Results show that post‐antibody treatment, AMs death decreases, with a reduction in weight loss, lower mortality rates, and alleviation of lung injury in mice. Unfortunately, the earliest clinical trials for treating sepsis with TNF‐specific antibodies did not yield the anticipated results [36]. However, newer findings suggest these antibodies might offer advantages in sepsis management, with efficacy depending on the choice of therapeutic agent and timing of administration postinfection [37, 38]. Additionally, while it is now clear that TNF binding to TNFR1 also promotes inflammation by inducing cell death forms like apoptosis, necroptosis, or pyroptosis, validating this function with specific cell death inhibitors remains challenging. Overall, the TNF‐α pathway continues to be a significant research area, offering potential insights into novel therapeutic approaches for various inflammatory and autoimmune disorders.

## 5 Conclusion

In summary, our research uncovers the pivotal role of monocyte‐derived macrophages in promoting alveolar macrophages death via TNF‐α during acute lung injury. This discovery suggests TNF‐α inhibitors as a potential therapeutic target for ALI/ARDS and the prospect of regulating inflammatory cell death in treating acute lung inflammation.

## Author Contributions

**Junjie Xiao:** conceptualization (lead), methodology (equal), data curation (lead), formal analysis (lead), investigation (equal), project administration (equal), visualization (lead), validation (equal), writing–original draft preparation (lead). **Fei Hou:** conceptualization (lead), methodology (equal), data curation (supporting), formal analysis (supporting), investigation (equal), project administration (equal), validation (equal), writing–review and editing (equal), supervision (equal). **Huan Wang:** software (lead), methodology (supporting), writing–review and editing (supporting). **Ruixuan Wang:** formal analysis (supporting), project administration (supporting), validation (supporting). **Ying Liu:** software (supporting), visualization (supporting), project administration (supporting). **Xiayan Wu:** project administration (supporting), formal analysis (supporting). **Lixin Xie:** conceptualization (supporting), funding acquisition (lead), project administration (equal), resources (lead), supervision (equal), writing–review and editing (equal).

## Ethics Statement

All animal experiments were conducted in accordance with the China Council on Animal Care and Use. This study has obtained the approval from Animal Care and Use Committee of the university (IACUC‐20200221‐02MO), and the animal experiments in our study were conducted in State Key Laboratory of Medical Proteomics, Beijing. Every effort was dedicated to minimizing the pain and discomfort to the experimental animals.

## Conflicts of Interest

The authors declare no conflicts of interest.

## Supporting information

Supporting information.

## Data Availability

The analyzed data sets generated during the study are available from the corresponding author on reasonable request.
