# Supplementary material for: Monocyte‐Derived Macrophages Induce Alveolar Macrophages Death via TNF‐α in Acute Lung Injury
Source: Immun Inflamm Dis. 2024 Dec 11;12(12):e70081. doi: 10.1002/iid3.70081 (PMC11632899; doi:10.1002/iid3.70081)
Supplement: Supplementary file 1 — Supporting information. [file IID3-12-e70081-s001.docx]

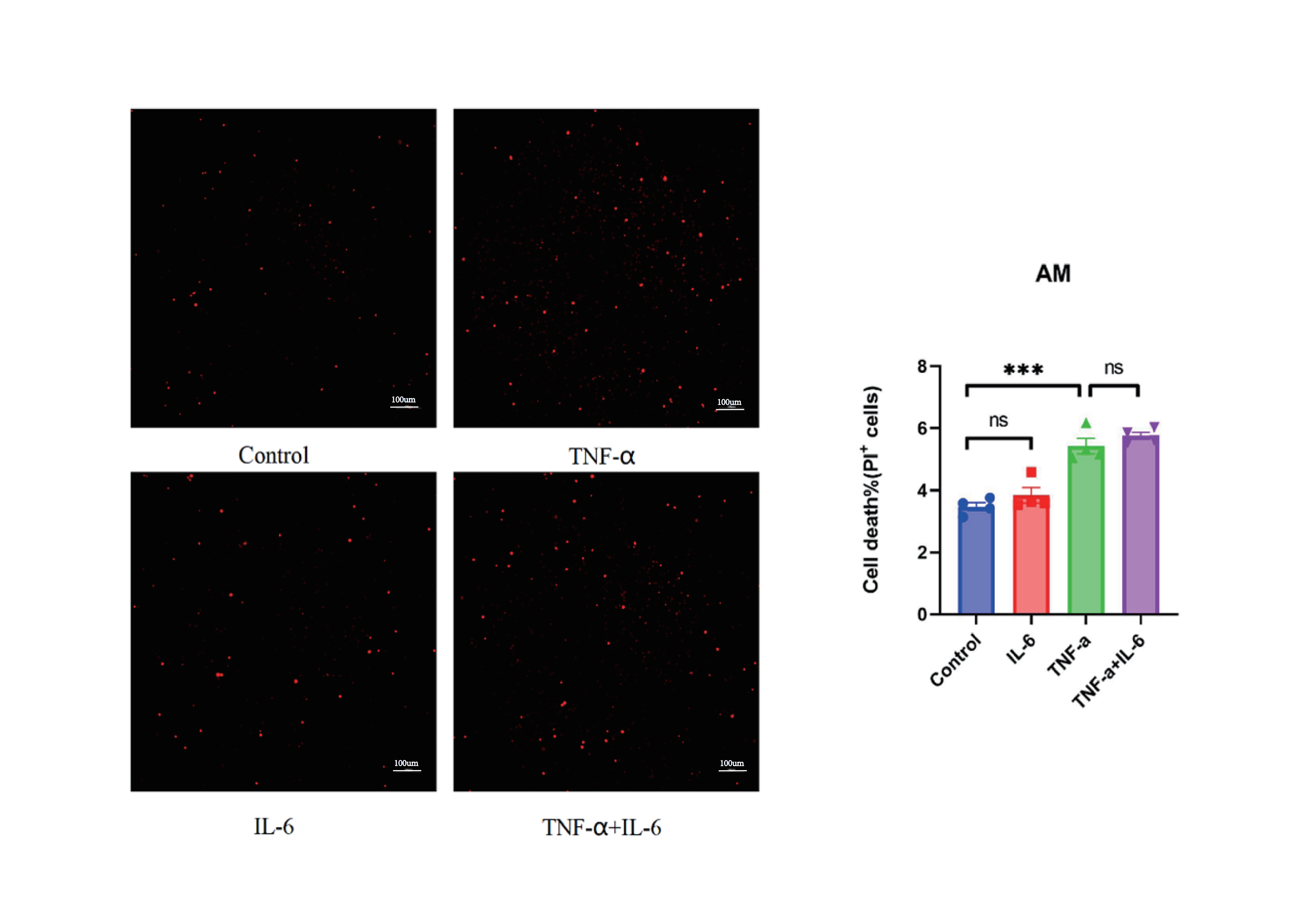


**Supplementary Figure 1.** **Representative images of cell death in AMs treated with TNF-α and IL-6 after 48 h.** AMs (3 × 105 cells/well) were seeded in 48-well tissue culture plates. Cells were treated with 200 ng /mL of TNF-α, 200 ng /mL of IL-6 or their combination and stained with 2.5 µg/ml of propidium iodide. The plate was scanned for fluorescent and phase-contrast images (4 image fields/well) after 48 h. Scale bar,100µm. Data are presented as mean ± SEM. ns, not significant, ***P < 0.001. Analysis was performed using Student’s t-test.
